# Predicting the Potential Distribution of *Cheirotonus jansoni* (Coleoptera: Scarabaeidae) Under Climate Change

**DOI:** 10.3390/insects15121012

**Published:** 2024-12-20

**Authors:** Yali Yu, Zhiqiang Li

**Affiliations:** Guangdong Key Laboratory of Animal Conservation and Resource Utilization, Guangdong Public Laboratory of Wild Animal Conservation and Utilization, Institute of Zoology, Guangdong Academy of Sciences, Guangzhou 510260, China; yuyl@giz.gd.cn

**Keywords:** *Cheirotonus jansoni*, Biomod2, MaxEnt, species distribution model, potential distribution

## Abstract

*Cheirotonus jansoni* (Jordan, 1898), a beetle species of ecological and ornamental significance, is predominantly found in southern China. With limited dispersal ability, it is classified as a Class 2 protected species in China. In this study, the widely employed maximum entropy (MaxEnt) model and the ensemble Biomod2 model were applied to simulate *C. jansoni* habitat suitability in China under current environmental conditions based on available distribution data and multiple environmental variables. The optimized MaxEnt model demonstrated improved accuracy and robust predictive capabilities, making it the preferred choice for simulating dynamic changes in potentially suitable habitats for *C. jansoni* under future climate scenarios. Protection gaps were further identified through analyses of the overlap between nature reserves and highly suitable areas for *C. jansoni*. The established models indicated that this species primarily resides in southeastern mountainous regions of China below 2000 m, with a preferred altitude of 1000–2000 m. Future climate scenarios suggest a reduction in the overall suitable habitat for *C. jansoni* with an increase in temperature, underscoring the urgent need for enhanced conservation efforts for this beetle species.

## Simple Summary

Global climate change is a key determinant of species distributions. Beetles, being a diverse and widely distributed group, demonstrate high sensitivity to environmental changes. This study modeled and predicted the distribution of the large-sized scarabaeid *Cheirotonus jansoni*, a species with a restricted range, under current conditions and two projected future climate scenarios. The relative importance of environmental factors responsible for regulating the distribution of this species was assessed, speculating that *C. jansoni* tends to prefer warmer, more humid, and stable habitats. The analyses revealed a reduction in suitable habitat for *C. jansoni*, particularly at lower latitudes under future warming scenarios, indicating the potential loss of this species in some regions. Altitude, in addition to latitude, was identified as a key environmental factor influencing the geographical distributions of *C. jansoni*. The analysis of potential *C. jansoni* distribution patterns in China, along with the identification of factors limiting its range, may provide a robust evidence base for the conservation and effective management of this species.

## 1. Introduction

The Sixth Assessment Report of the Intergovernmental Panel on Climate Change suggests that the past 50 years, since 1970, represent the warmest half-century in the last two millennia, with current global average temperatures being 1.1 °C above the average temperature from 1850–1900 during the Industrial Revolution and continuing to rise. Global temperature increases are predicted to exceed 1.5 °C within the next 20 years [1]. Beyond this threshold, there is a risk of the temperature crossing multiple tipping points, leading to significant impacts, including the dieback of biodiverse biomes such as the Amazon rainforest or warm-water corals [2].

Range shifts are an area of active research interest in studying the effects of climate change on different species [3,4]. Global climate change is predicted to cause widespread alterations in surface temperatures and precipitation patterns worldwide [5,6,7], significantly affecting biodiversity and ecosystems [8,9]. Furthermore, climate change may have a significant effect on insects [10,11,12]. The ability of insects to adapt to changing climate is determined by the rate of climate change, the biological characteristics of a given species, and the rate at which these species can modify their traits in response to the changing environment [13]. These factors reduce the likelihood that most species will effectively adapt to the ongoing climate change [11]. The shrinking, degradation, and loss of habitats attributable to climate change represent a growing threat to the distribution of insects and other populations, potentially accelerating the extinction of species. Such risks are higher for species with highly patchy or discontinuous habitats, as well as those with a limited range and the lack of ability to spread readily [11,14,15]. Research studies focused on species’ responses to future climatic change can contribute to clarifying the factors that give rise to species’ geographical distributions while also assisting the formulation of evidence-based germplasm resource management strategies.

Species distribution models (SDMs) are valuable tools for research focusing on the effects of environmental and climate change on suitable areas for species [16]. They are extensively employed in conservation biology to predict potential shifts in species distributions under different conditions [17]. These models can provide a strong theoretical foundation for the delineation of protected areas for certain endangered species, supporting ex situ protection efforts and management decision-making [18].

Recent studies on insect conservation have increasingly relied on SDMs to predict habitat changes [12,15,19]. Rapid advances in information technologies have driven the emergence of new modeling algorithms, enhancing the ability to forecast species distributions under different environmental scenarios [20]. Maximum entropy modeling (MaxEnt) is among the most commonly employed such models, but its accuracy is greatly influenced by selected model parameters and environmental variables [21]. By combining the MaxEnt model with R, model performance can be enhanced through the adjustment of parameters, resulting in a model with improved predictive accuracy. In recent years, optimized MaxEnt models have become a widely employed tool for predicting suitable habitats, offering improved accuracy in assessing species distribution [22,23,24]. However, Thuiller [25] suggested that single models show fluctuating performance with alterations in the input data, yielding relatively poor stability. The integration of results derived from more than one model may provide an effective means of improving predictive accuracy. The R-based Biomod model platform can also be used for this purpose. Each model employs characteristic principles and algorithms associated with various strengths and limitations [26]. When the same model is used for different species, it may differ substantially in its predictive precision, while using different models with the same species may yield inconsistent predictions of suitable habitats [27]. The assessment of model performance and the identification of optimal models are essential aspects of research, particularly in enhancing the accuracy and reliability of species distribution predictions [27]. Known distribution data were used to construct various models while also accounting for spatial bias associated with modeling approaches, followed by the comparison of differences among individual and combined models. This approach provides valuable insights that can guide future studies in selecting appropriate SDMs for similar modeling contexts.

*Cheirotonus jansoni* (Insecta: Coleoptera: Scarabaeidae) is a rare species of beetle and a Class 2 protected wildlife in China. Its distribution area includes Jiangsu, Anhui, Zhejiang, Hubei, Jiangxi, Hunan, Fujian, Guangdong, Hainan, Guangxi, Chongqing, Sichuan, and Guizhou in China [28,29,30,31,32]. *Cheirotonus jansoni* feeds on rotting wood and humus and is an important decomposer to the circulation of materials within forest ecosystems. *Cheirotonus jansoni* is characterized by its large size and exceptionally long prolegs, which hinder its mobility [32]. The male’s long prolegs play a crucial role in mating behavior. Males often guard the hole in the tree and stretch their long prolegs into the tree hole to entice the females to leave the “boudoir”. During mating, the long prolegs can be used to hold the female firmly, making it difficult for the females to escape [29,33]. The long prolegs, while beneficial for mating, present challenges for mobility. It is easy to encounter obstacles such as branches when they are flying. Once they fell, it could be difficult to turn over due to their large size [29,30]. Excessive collection driven by its ornamental value has led to a significant decline in the *C. jansoni* population [29,31] and it was declared extinct in China in 1982 [30]. More recently, however, individuals from this species have been discovered in areas including Guizhou, Chongqing, and Jiangxi (see the Global Biodiversity Information Facility: GBIF, https://www.gbif.org/, accessed on 31 October 2023). There is an urgent need for further research and conservation efforts for *C. jansoni*, as few studies have been conducted, mainly focusing on morphological characterization, discovery reports, habitat observations, and mitochondrial genome analyses.

In this study, SDMs were used to estimate the potential distribution of *C. jansoni* in China based on current climatic conditions and two future climate scenarios. The main objectives of the study included the following: (1) to estimate the relative importance of environmental factors influencing the distribution pattern of *C. jansoni*; (2) to predict the potential distribution of *C. jansoni* under climate change; (3) to assess the effectiveness of currently protected areas in China by comparing them with the potential distribution of *C. jansoni*. The findings will provide insights into the environmental factors limiting the potential geographical distribution of this beetle species, offering a scientific evidence base for conserving and managing the germplasm resources for this species.

## 2. Materials and Methods

### 2.1. Occurrence Records

**Figure 1:**
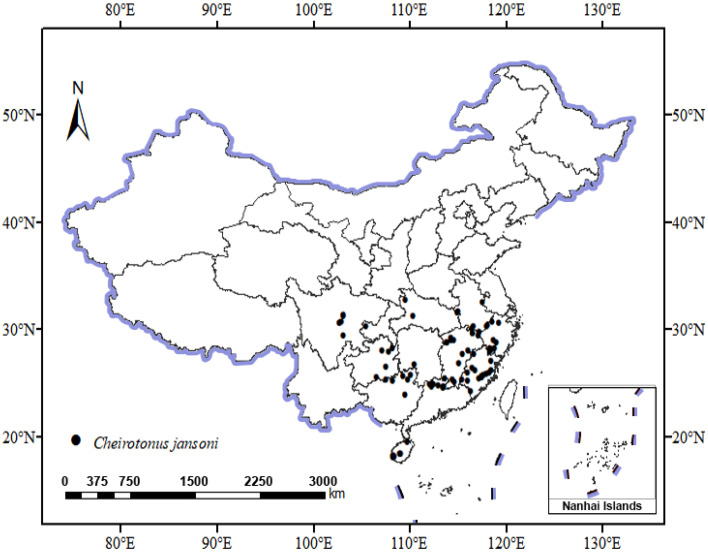
Recorded locations of *C. jansoni* in China (1925–2023).

Occurrence records of *C. jansoni* were obtained from the Global Biodiversity Information Facility (GBIF, https://www.gbif.org/, accessed on 31 October 2023), National Specimen Information Infrastructure (NSII, http://www.nsii.org.cn/, accessed on 20 April 2024), relevant publications [28,29,30,31,32], as well as unpublished records from public observation databases and fieldwork. Subsequent analyses were based on distribution data with accurate longitudes and latitudes, excluding redundant data or suspicious data. Latitudes and longitudes for records lacking precise geo-coordinates were confirmed through coordinate selection in Baidu maps. To minimize the potential for model overfitting due to species distribution clustering effects, spatial autocorrelation analyses were conducted with the Perl script-based ENMTools (version 1.0.4), retaining just one distribution point per 5 km × 5 km grid. In total, 90 occurrence records with strong geo-coordinates were retained for modeling. The observed data appertaining to *C. jansoni* distributions in China were compiled in a map (Figure 1). Map data were derived from the National Platform for Common GeoSpatial Information Services (https://www.tianditu.gov.cn/, accessed on 10 May 2024).

### 2.2. Environmental Variables

Climate data for this study included 19 bioclimatic variables (Table S1) related to the current and future climate, all downloaded from the Worldclim database (v 2.1; http://worldclim.org, accessed on 30 November 2023) at a spatial resolution of 2.5 arc-minutes. Current climate data were based on records of monthly temperature and precipitation from 1970–2000, while future climate projections were derived from the parallel Shared Socioeconomic Pathways (SSPs) and the Beijing Climate Center Climate System Model (BCC-CSM2-MR) data [34]. Predictions were made for two future climatic scenarios (SSP245 and SSP370), focusing on four timelines (2021–2040, 2041–2060, 2061–2080, and 2081–2100). SSP245 represents an intermediate development pathway with moderate radiative forcing, stabilizing at 4.5 W/m^2^ in 2100. SSP370, however, focuses on a regional competitive development path with a stable radiative forcing of 7.0 W/m^2^ in 2100. Higher radiative forcing values correspond to more rapid climate warming. During the period 2081–2100, the global annual mean surface air temperature is projected to increase by approximately 2.0 °C under SSP245 (relative to 1995–2014), while under SSP370, the global annual mean surface air temperature is anticipated to increase by about 3.1 °C [35].

**Figure 2:**
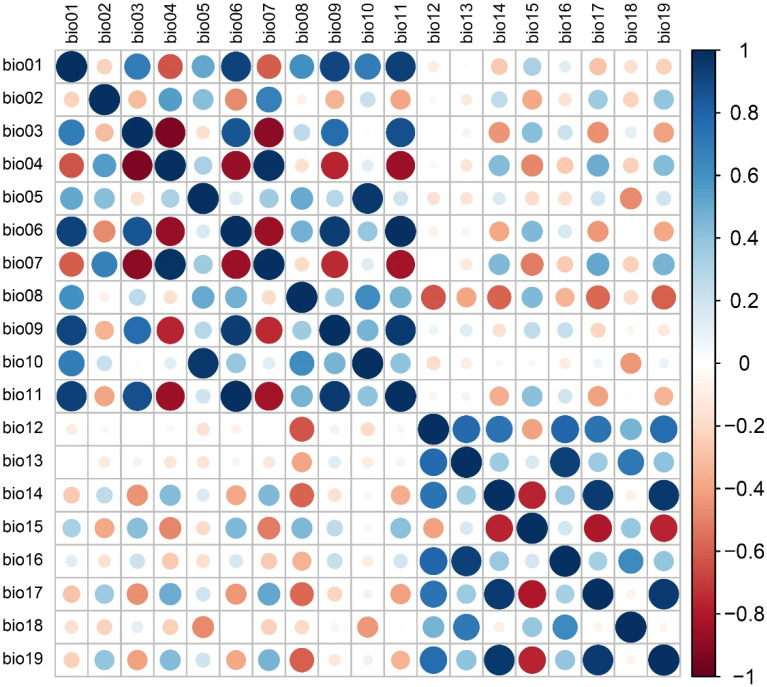
Pearson correlation matrix for individual environmental variables. Red circles and blue circles are respectively used to depict negative and positive correlations. The larger the size of circles, the stronger the relationship between two variables.

The “extract by mask” function in ArcGIS was used to clip the bioclimatic layers to match study area dimensions, and the observed results were saved in an ASCII grid format for subsequent analyses. To prevent the overfitting of model predictions due to environmental variable collinearity and to enhance predictive accuracy [36], the Jackknife test, percent contribution, the permutation importance in MaxEnt values [37], and Pearson analyses in SPSS [38] were used to evaluate the importance and correlations among environmental variables (Figure 2). Variables with ≤1% contribution rates in the initial models were excluded from further analysis. Furthermore, those variables exhibiting correlations |r| < 0.8 were screened [23,37], with one variable removed when two highly correlated variables were identified. Ultimately, eight environmental variables were retained for analysis (Table S1).

### 2.3. Modeling Strategy

The maximum entropy (MaxEnt, version 3.4.1) model was selected for this study due to its extensive use in predicting species distributions in recent studies [39,40]. The performance of this model can be affected by both the feature class (FC) and regularization multiplier (RM) [21]. The MaxEnt model incorporates five features: linear (L), quadratic (Q), fragmented (H), product (P), and threshold (T) features. To prevent model overfitting while enabling optimal model selection, the R Kuenm package version 1.1.10 was employed for the optimization of these parameters [41]. A total of 1240 candidate models were evaluated, incorporating parameters that corresponded to all combinations of 40 RM settings (ranging from 0.1 to 4, with an interval of 0.1) and 31 FC combinations. Statistical significance was used to guide model selection, together with predictive ability (low omission rates) and complexity (AICc values). These considerations were prioritized by initially omitting non-significant models, followed by retaining only those models that met the omission rate criterion (<5% when possible). The remaining models were then selected based on the lowest delta AICc (Akaike information criterion) values, with a threshold of <2 [41,42,43]. A random test percentage of 25% was selected, utilizing 10 replicates to obtain a model average while leaving the remaining values at default settings. The performance of the MaxEnt model fit was evaluated using the area under the curve (AUC) from the receiver operating characteristic (ROC) curve [44].

Ensemble forecasting model construction was performed using the R (version 4.2.3) Biomod2 package (version 3.5.1), allowing for the training of SDMs with different modeling approaches, model evaluation, and producing outputs in the form of single-model predictions [45]. This study was based on considering 10 modeling algorithms for the generation of potential distributions, including generalized linear models (GLMs), generalized boosted models (GBMs), classification tree analysis (CTA), generalized additive models (GAMs), surface range envelope modeling (SRE), artificial neural networks (ANNs), flexible discriminant analysis (FDA), multivariate adaptive regression splines (MARSs), random forest (RF), and MaxEnt models. For the modeling process, the distribution data were randomly divided into a training dataset, comprising 75% of the samples, and a testing dataset, comprising the remaining 25%. The described process was repeated twice to ensure robustness and reliability in the model’s performance. To provide better simulations of the actual distribution while reducing spatial deviation, 1000 pseudoabsence points were randomly selected, with the process being repeated twice for model construction. This approach resulted in the establishment of 40 SDMs. Model evaluation was performed based on AUC, Kappa [46], and true skill statistics (TSS) values [47]. The ensemble model (EM) was developed by selecting individual models with a TSS ≥ 0.8 for simulating potential distribution areas. Two methods were used for ensemble construction: the weighted mean of probabilities (wm) and committee averaging (ca), with the latter providing equal weight to all predictions [45].

### 2.4. Distributional Dynamic Changes and Centroid Location

The optimized MaxEnt model estimates suitable habitats for *C. jansoni* on a scale from 0 to 1, with 0 representing low suitability and 1 representing high suitability, generating a response curve for each of the analyzed environmental variables. The model results were transformed using ArcGIS. Results were classified with the Maximum Test Sensitivity Plus Specificity (MTSPS) threshold method, which divided the spatial regions into two categories. Regions with species existence probabilities at or above the MTSPS threshold were classified as suitable areas, while those below the threshold were categorized as unsuitable. The reclassification of suitable areas into three classes was also performed, including areas of low suitability (MTSPS < probability values ≤ 0.4), moderate suitability (0.4 < probability values ≤ 0.6), and high suitability (probability values > 0.6) [48]. To enable better comparisons of shifts in potentially suitable areas for *C. jansoni* between the present and future, the number of grids in suitable and unsuitable areas was calculated for both the current and future scenarios using the “Raster Calculator” function in ArcGIS.

Distributional changes were quantified using SDMtoolbox, calculating shifts in the geometric centroid of potentially suitable areas for *C. jansoni*. Future changes in area were assessed based on current suitable zones, with spatial pattern changes scored as follows: −1 for range expansion, 0 for no occupancy (absence in both scenarios), 1 for no change (presence in both scenarios), and 2 for range contraction.

### 2.5. Conservation Gaps

The “extract by mask” function in ArcGIS was used to clip these provinces with highly suitable areas for *C. jansoni* from the map of China. Following this, highly suitable areas for *C. jansoni* and the nature reserves of China were displayed in the same view. Following this, the overlap of the national nature reserves and highly suitable areas for *C. jansoni* was evaluated. Finally, the conservation gaps were identified from the visualized figures.

## 3. Results

### 3.1. Model Performance

**Figure 3:**
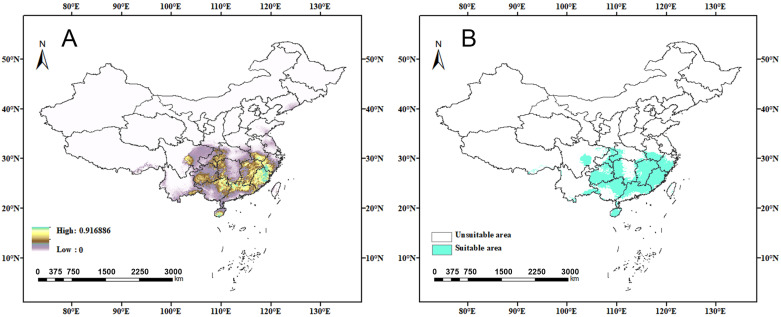
Predicted *C. jansoni* geographic distributions under current climatic conditions based on optimized MaxEnt spatial distribution modeling approaches. (**A**) Continuous suitability values; (**B**) Habitats classified into two grades.

Based on 90 distribution records for *C. jansoni* and eight environmental variables (see Table S1), an optimized MaxEnt model and an ensemble Biomod2 model were developed to simulate and predict suitable areas for *C. jansoni*. Models utilizing optimal parameters (RM = 0.2, FC = LQ) outperformed the default models (RM = 1, FC = LQHPT), with a delta AICc = 0. The habitat suitability assessed by the optimized MaxEnt model under current climatic conditions closely aligned with the observed distribution of *C. jansoni* (Figure 3), achieving strong modeling performance (AUC = 0.959, Figure S1).

**Figure 4:**
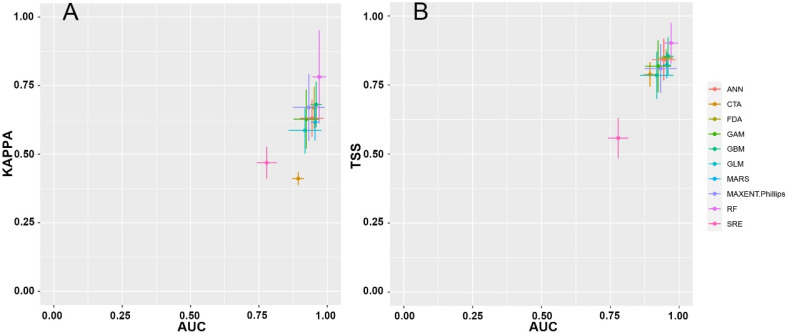
Evaluation parameters for single Biomod2 models based on 10 modeling algorithms. (**A**) ROC and Kappa; (**B**) ROC and TSS.

**Figure 5:**
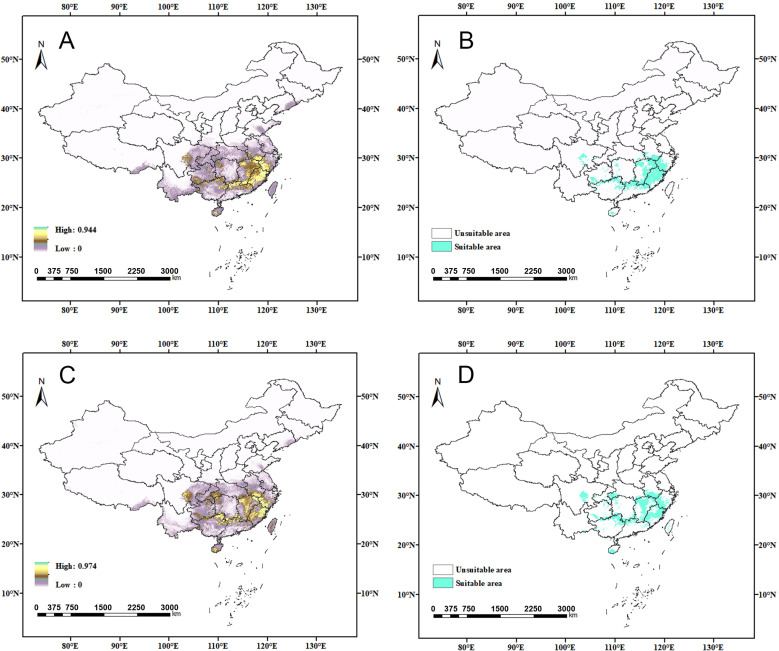
Potential predicted geographic distributions of *C. jansoni* based on current climatic conditions for the RF spatial distribution modeling strategies. (**A**–**D**) Predictions with the first (**A**,**B**) and second (**C**,**D**) selection of pseudoabsence points, with suitability shown as continuous (**A**,**C**) or classified into two grades (**B**,**D**).

**Figure 6:**
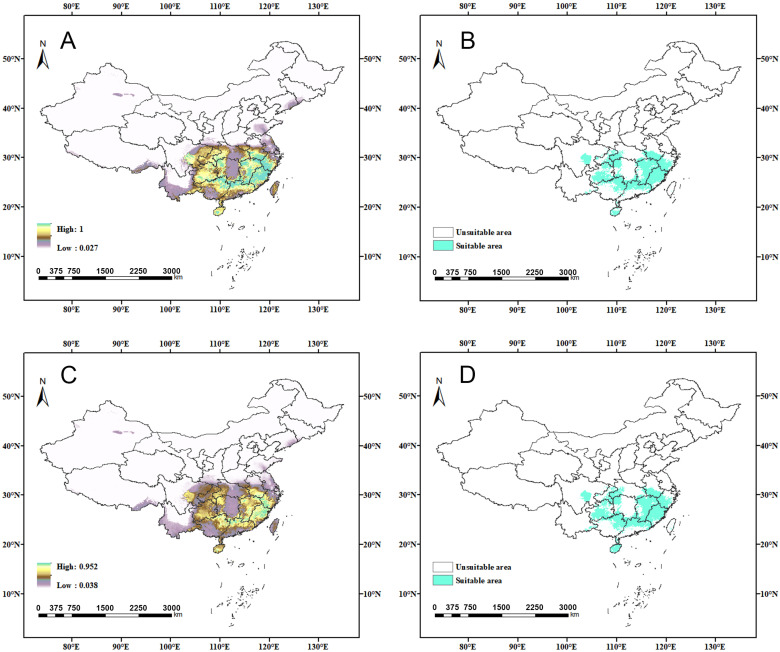
Potential *C. jansoni* geographic distributions under current climatic conditions based on the ensemble spatial distribution modeling approaches. (**A**–**D**) Predictions when the committee averaging method (**A**,**B**) or weighted mean of (**A**–**D**) Predictions using the committee averaging method (**A**,**B**) or the weighted mean of probabilities method (**C**,**D**), with suitability shown as continuous (**A**,**C**) or classified into two grades (**B**,**D**).

Individual Biomod2 models based on 10 different algorithms showed variations in evaluation parameters (Figure 4, Table S2) and predicted distribution regions. Among these, the RF model demonstrated relatively high evaluation values and appeared to be well aligned with the actual distribution of *C. jansoni* (Figure 5). However, the RF model predicted a significantly narrower suitable area compared to other models, suggesting an overly conservative estimate of the potential distribution. Ensemble models developed by selecting individual models with a TSS ≥ 0.8 showed relatively strong evaluation metrics (Table S2). However, some of these models provided unreasonable predictions regarding the species’ distribution areas (Figure 6). Through comparative analyses of evaluation parameters and consistency with actual *C. jansoni* distributions, the MaxEnt model, adjusted with the Kuenm package, was selected for subsequent future modeling.

### 3.2. Response of Environmental Variables

Of the eight variables used to construct the optimized MaxEnt model (Table S1), the three most significant predictors of suitable areas for *C. jansoni* were average variation in daytime temperature (bio02), contributing 53.9%, followed by annual precipitation (bio12) at 14.7% and precipitation during the hottest quarter (bio18) at 10.8%. The influence of the remaining variables was comparatively limited. All of these environmental variables contributed to the model to some extent, confirming the rational basis for their selection. Figure S2 shows the Jackknife results for individual environmental variables in this optimized MaxEnt model. Six variables (bio02, bio10, bio12, bio13, bio14, bio18) displayed relatively high values (>0.4) when considered independently, indicating they capture more critical information. However, the contributions of bio3 and bio8 in isolation were relatively minor, indicating that these are less informative environmental factors.

**Figure 7:**
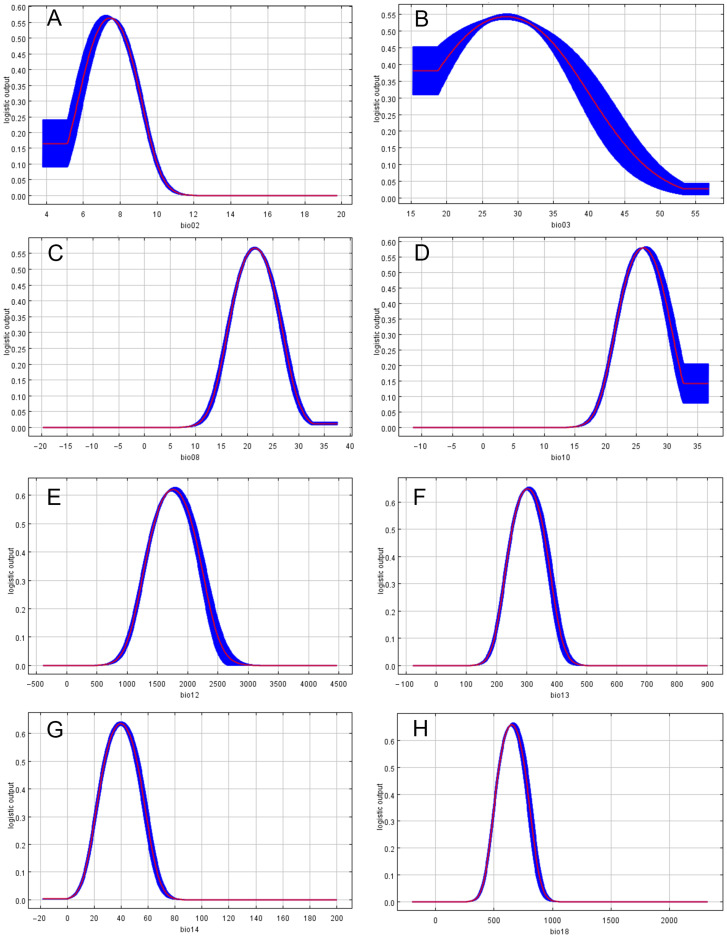
Environmental variable response curves under the optimized MaxEnt model demonstrating the relationships between the probability of *C. jansoni* existence and eight different environmental variables. In each panel, the X- and Y-axes correspond to environmental variables and the presence probability of species, respectively. The curves demonstrate the means responses from 10 MaxEnt replicate runs. (**A**) Daytime temperature (bio02); (**B**) Isothermality (bio03); (**C**) Average temperature during the rainiest quarter of months (bio08); (**D**) Average temperature during the hottest quarter of months (bio10); (**E**) Annual precipitation (bio12); (**F**) Precipitation during the rainiest month (bio13); (**G**) Precipitation during the driest month (bio14); (**H**) Precipitation during the hottest quarter of months (bio18).

Logistic regression analyses were used to explore the relationships between environmental variables and the distribution probabilities for suitable areas of *C. jansoni* in the MaxEnt model, enabling the construction of single-factor response curves (Figure 7). According to the “average Maximum test sensitivity plus specificity Logistic threshold”, suitable areas were identified as regions with a geographic distribution probability exceeding 0.1671. These results suggest that *C. jansoni* tends to prefer areas with the following environmental conditions: an average variation in daytime temperature (bio02) ranging from 5.2 °C to 9.7 °C, with 7.4 °C being most optimial (Figure 7A); isothermality (bio03) less than 44.8, with 28.2 being optimal (Figure 7B); an average temperature during the rainiest quarter of months (bio08) from 14.5 °C to 28.5 °C, with 21.4 °C being optimal (Figure 7C); an average temperature during the hottest quarter of months (bio10) from 20.0 °C to 32.3 °C, with 26.2 °C being optimal (Figure 7D); annual precipitation (bio12) from 1115.0 mm to 2404.5 mm, with 1762.1 mm being optimal (Figure 7E); precipitation during the rainiest month (bio13) from 205.6 mm to 402.7 mm, with 303.7 mm being optimal (Figure 7F); precipitation during the driest month (bio14) from 16.1 mm to 63.05 mm, with 39.9 mm being optimal (Figure 7G); precipitation during the hottest quarter of months (bio18) from 450.3 mm to 857.9 mm, with 652.8 mm being optimal (Figure 7H).

### 3.3. Current Potentially Suitable Areas

Figure 3 presents the suitable areas for *C. jansoni* in China based on current climatic conditions. The total suitable area for *C. jansoni* was 1044.19 × 10^3^ km^2^, accounting for 10.88% of China’s total land area. Of this area, an estimated 609.47 × 10^3^ km^2^ (6.35% of total land area) had low suitability, 298,920 km^2^ (3.11%) had moderate suitability, and 135,800 km^2^ (1.41%) had high suitability.

The estimated distribution range of *C. jansoni* in China ranges approximately 92.0–121.3° E and 18.3–34.0° N, covering central, eastern, southern, and southwestern portions of China. The species is predicted to be primarily found in Jiangxi, Fujian, Guizhou, Guangxi, Guangdong Province, and Hainan Island, with a relatively limited distribution in Sichuan, Zhejiang, Hunan, Hubei, Chongqing, Anhui, Yunnan, and other provinces.

The highly suitable distribution areas were dispersed, with a larger continuous region primarily observed in Fujian and Zhejiang Provinces. The moderately suitable distribution areas largely mirrored the highly suitable areas, extending outwards to some degree with the highly suitable areas in the center. A similar pattern was observed for areas of low suitability.

### 3.4. Future Potentially Suitable Areas

**Figure 8:**
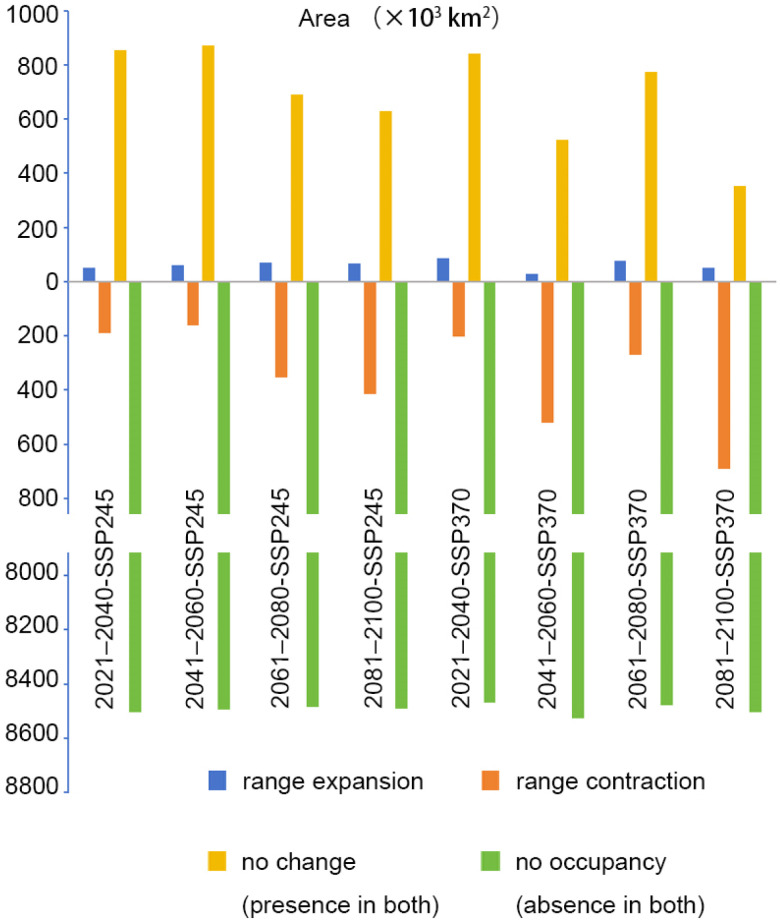
Changes in the range of *C. jansoni* based on binary distributions across different periods and climate change scenarios relative to current conditions (units: 10^3^ km^2^).

**Figure 9:**
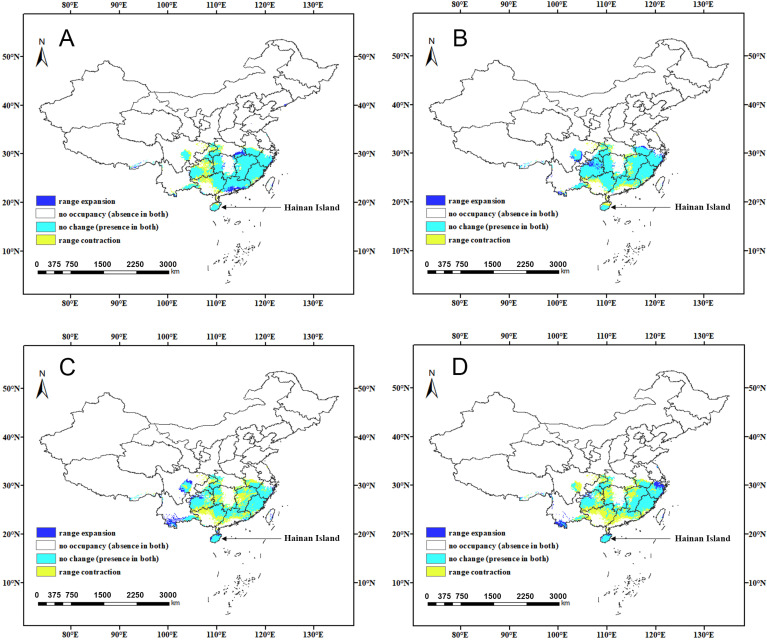
Changes in suitable areas of China for *C. jansoni* under future climate scenarios: (**A**) 2021–2040-SSP245; (**B**) 2041–2060-SSP245; (**C**) 2061–2080-SSP245; and (**D**) 2081–2100-SSP245.

**Figure 10:**
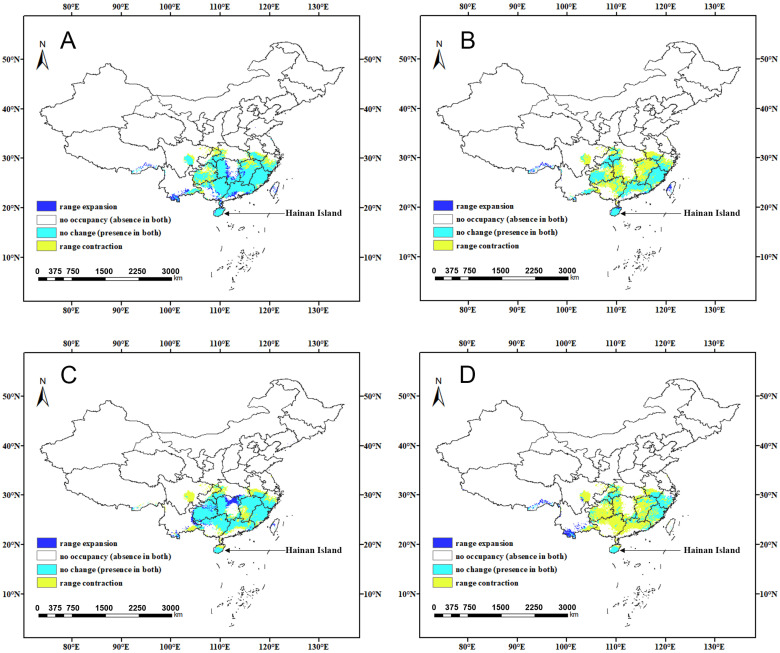
Changes in suitable areas of China for *C. jansoni* under future climate scenarios: (**A**) 2021–2040-SSP370; (**B**) 2041–2060-SSP370; (**C**) 2061–2080-SSP370; and (**D**) 2081–2100-SSP370.

The spatial patterns of suitable areas for *C. jansoni* under current climatic conditions were compared with those predicted for future climate scenarios (Table S3 and Table S4, Figure 8, Figure 9 and Figure 10). Compared to the current climate conditions, the retention rate for suitable areas for *C. jansoni* was found to be below 85%. Under both future climate scenarios, the areas of expansion for *C. jansoni* in China were consistently smaller than the areas of contraction, highlighting both ongoing and predicted future reductions in the total suitable habitat for this species.

Predictions of suitable area expansion and contraction of suitable areas for *C. jansoni* varied depending on the timeframe and climate scenario. The rate of suitable area expansion ranged from 2.7 to 8.4%, with increases in total area from 28.37 × 10^3^–87.95 × 10^3^ km^2^ under the two future climate scenarios. The lowest and highest expansion rates were, respectively, observed under the 2041–2060-SSP370 and 2021–2040-SSP370 climate scenarios, with range expansions predominantly occurring in parts of Yunnan and Guizhou. Contraction rates for suitable areas ranged from 15.9 to 66.3%, with decreases in total area from 166.03 × 10^3^–691.8 × 10^3^ km^2^ under the two future climate scenarios. The respective lowest and highest contraction rates were observed under the 2041–2060-SSP245 and 2081–2100-SSP370 climate scenarios, respectively. Guangdong and Guangxi were the regions most frequently exhibiting range contraction.

### 3.5. Centroid Migration

**Figure 11:**
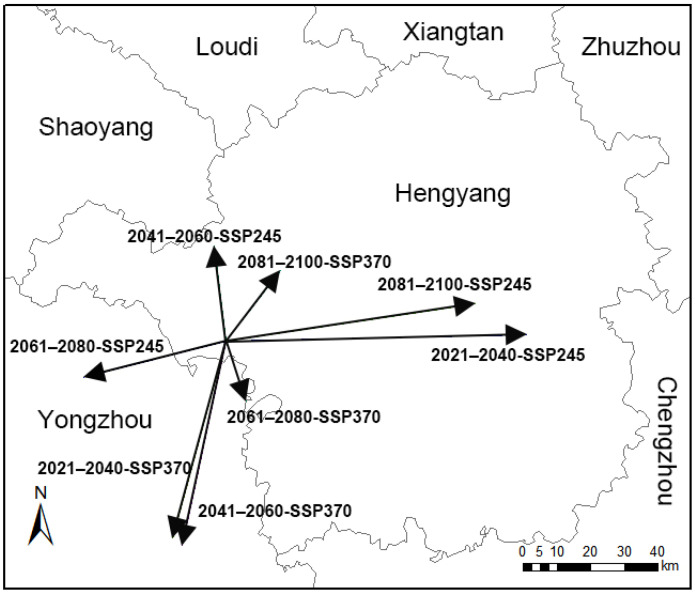
Changes in the centroid of potential distributions for *C. jansoni* under various climate scenarios. Arrows represent the direction and magnitude of core distributional shifts with time.

Centroids were selected to represent the overall spatial positions of potentially suitable areas for *C. jansoni* under current climatic conditions (Figure 11). Currently, the centroid of the current potential suitable area of *C. jansoni* is located in Hengyang city (112.09° E, 26.70° N) in Hunan Province.

The geometric centroids for areas potentially suitable for *C. jansoni* during 2041–2060-SSP245, 2081–2100-SSP370, 2081–2100-SSP245, and 2021–2040-SSP245 were all found in Hengyang city, while those for 2061–2080-SSP245, 2021–2040-SSP370, 2041–2060-SSP370, and 2061–2080-SSP370 were situated in Yongzhou City in Hunan Province.

The migration distances of the predicted centroids for *C. jansoni* under the SSP245 scenario relative to current conditions were 77,808 m (2021–2040), 29,586 m (2041–2060), 38,847 m (2061–2080), and 65,582 m (2081–2100). In comparison, the corresponding values for SSP370 and current scenarios were found to be 62,265 m, 63,850 m, 19,320 m, and 26,014 m, respectively.

### 3.6. Conservation Gaps

**Figure 12:**
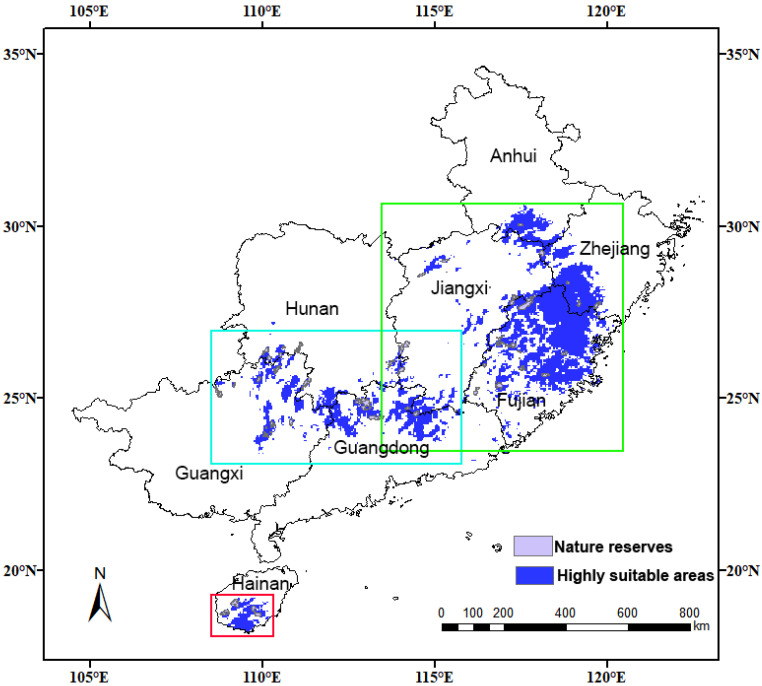
Nature reserves of China in highly suitable areas for *C. jansoni.* Areas in the green box include Anhui, Zhejiang, Jiangxi, and Fujian Provinces. Areas in the blue box include Hunan, Jiangxi, Guangxi, and Guangdong Provinces. Areas in the red box include Hainan Island. Further details are provided in Figure 13.

**Figure 13:**
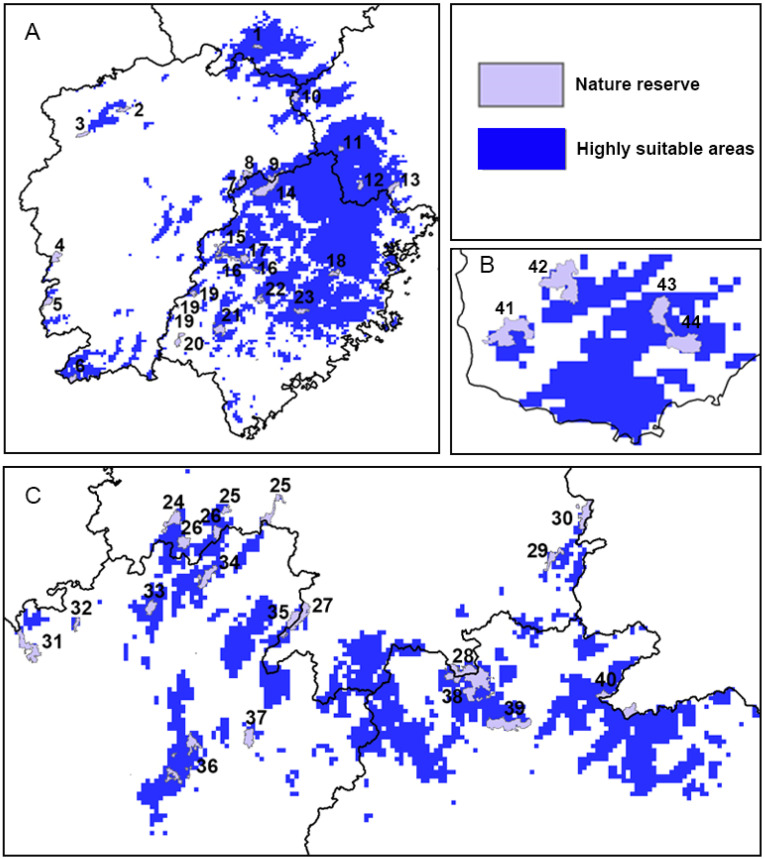
National nature reserves of China in highly suitable areas for *C. jansoni.* (**A**) Anhui, Zhejiang, Jiangxi, and Fujian Provinces; (**B**) Hainan Island; (**C**) Hunan, Guangxi, and Guangdong Province. Numbers denote the national reserve numbers presented in Table S4.

An overlap analysis between over 400 national nature reserves in China and highly suitable areas for *C. jansoni* (Figure 12 and Figure 13; Table S4) revealed that 43 national nature reserves (10%) contained highly suitable habitats for the species. While highly suitable areas for *C. jansoni* cover just ~1% of the total area of China, they are concentrated within numerous national nature reserves, indicating a strong correlation between these protected areas and the species’ survival. In the majority of these national nature reserves, including Nanling, Wuyi Mountain, and Wuzhi Mountain, 100% of the area for the individual reserve was suitable for *C. jansoni.* However, substantial conservation gaps persist, particularly in northeastern Fujian, southern Hainan Island, and Guangdong, where suitable habitats lack effective protection.

## 4. Discussion

### 4.1. Optimal Model Selection

Selecting the optimal SDM remains challenging due to the diversity of available models, each with its applications and predictive strengths. MaxEnt is one of the most widely employed SDMs, with various previous studies relying on species distribution predictions generated using default model parameters. These analyses, however, are prone to overfitting and complexity, which may limit accuracy and produce results that are difficult to interpret [49]. Optimizing MaxEnt model parameters is thus vital to avoid overfitting and improve species distribution predictions [41]. To minimize error in the present study, 1240 combinations of parameters (40 RM settings and 31 FC combinations) were evaluated using the R Kuenm package. The results revealed that the optimal performance of the MaxEnt model was achieved with the settings FC = LQ and RM = 0.2.

However, Thuiller [25] suggested that the combination of prediction outputs from different models into an ensemble model can yield greater predictive performance [45,50]. Here, the ensemble Biomod2 model was therefore employed for the prediction and simulation of suitable areas for *C. jansoni*. The individual Biomod2 models were developed using 10 different modeling algorithms, while the Biomod2 ensemble model construction was achieved by selecting individual models with a TSS ≥ 0.8. Based on evaluation values and consistency with known distributions, the Kuenm package-optimized MaxEnt model was selected for subsequent modeling, as it provided accurate predictions of suitable habitats for *C. jansoni*. This optimized MaxEnt model is capable of effectively simulating *C. jansoni* geographical distributions with a high degree of reliability. When selecting an SDM, complexity does not necessarily equate to better performance. It is vital to critically compare models with actual input data to evaluate the most appropriate tool for a given context.

### 4.2. Environmental Suitability

Pearson and Dawson [51] suggested that abiotic factors, particularly climatic factors, primarily dominate large-scale species distribution and migration patterns. The current large-scale study focused on China, emphasizing the effects of climatic factors on the distribution of *C. jansoni*. The analyses revealed that average variation in daytime temperature (bio02), annual precipitation (bio12), and precipitation during the hottest quarter of months (bio18) were the primary environmental determinants of suitable areas for *C. jansoni*.

Response curves for environmental variables in the optimized MaxEnt model (Figure 7) suggested that *C. jansoni* likely prefers warm, humid, stable habitats. The highest probability of presence was observed when the average variation in daytime temperature (bio02) was 7.4 °C, the average temperature during the hottest quarter of months (bio10) was 26.2 °C, and the annual precipitation (bio12) was 1762.1 mm. Previous studies have demonstrated that *C. jansoni* predominantly inhabits tropical and subtropical evergreen broad-leaved forests or mixed evergreen-deciduous broad-leaved forests [52], consistent with the habitats identified as suitable for *C. jansoni*.

The large size of *C. jansoni* adults, coupled with their limited flight capabilities, prolonged larval stages, and reduced reproductive capacity, makes them particularly sensitive to environmental changes throughout their life cycles. Therefore, they are confined to inhabiting narrow ecological niches. Stable average daytime temperature variations are considered more favorable for the survival and thriving of *C. jansoni*. *Cheirotonus jansoni* larvae live in decaying wood and feed on humus, whereas adults subsist on tree sap. Moderate levels of moisture are assumed to be beneficial to *C. jansoni* development. Increased precipitation levels are considered advantageous for the survival of these beetles due to the correlation between precipitation and moisture.

**Figure 14:**
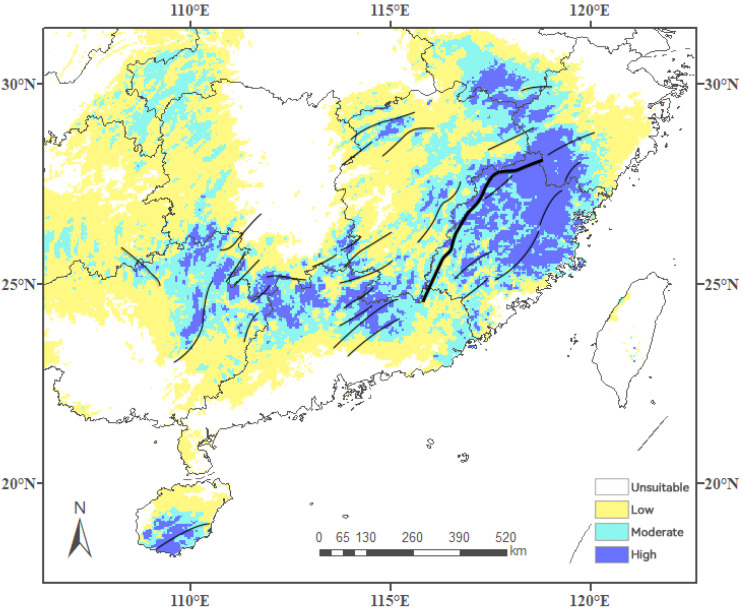
Major mountains in highly suitable areas for *C. jansoni*. Black lines within high suitability areas denote major mountains.

Through comparisons of the overlap between major mountains in China and highly suitable areas for *C. jansoni* (Figure 14), a close correlation was observed between the survival of this species and the mountains. Most of the mountains surrounding the highly suitable areas reach elevations of 1000–2000 m above sea level, consistent with the elevations where *C. jansoni* was found in our investigations. These mountains are located within the southeastern Mountain Region, offering diverse habitats with stable environmental conditions. Various mountainous areas in southeastern China, including the Nanling Mountains and Wuyi Mountains, have provided diverse habitats for species survival and maintenance [52,53].

### 4.3. Responses of Distribution Patterns to Climate Change

Beetles are highly sensitive to environmental changes and respond rapidly to these shifts in climatic conditions [54,55]. Various studies have explored the relationship between climate change and changes in beetle distribution patterns [11,37]. Comparisons of changing *C. jansoni* spatial distribution patterns under various future climate change scenarios revealed clear shifts in these patterns with environmental changes. These findings indicate that global warming is expected to adversely affect the growth and development of *C. jansoni* due to the anticipated reduction in their suitable habitat range. Over time, a more pronounced decline was observed in the suitable area for *C. jansoni* under the SSP370 climatic scenario compared to SSP225 (Figure 9D and Figure 10D). It indicates that larger temperature increases will lead to a more significant decline in the suitable area for *C. jansoni*. This aligns well with the findings of a previous study suggesting that the warming of the global climate will aggravate suitable habitat loss for various species [56]. With milder changes in temperature, species will tend to shift up to higher latitudes and elevations [57]. As temperatures continue to rise due to climate warming, *C. jansoni* is anticipated to experience significant habitat loss at lower latitudes, with the entire loss of some areas. However, Hainan Island represents an exception; despite its location at lower latitudes, future climate change is not predicted to significantly affect the range of *C. jansoni* in this region. As the second largest island in China, Hainan Island is influenced by a tropical monsoon climate with high volumes of precipitation and warm, humid conditions [58]. Therefore, marine islands may be an important factor influencing the persistence of beetle species due to their slower biotic turnover and higher thermal stability compared to continental lands [37,59]. Further, with warming climate conditions, suitable areas for *C. jansoni* are predicted to expand in the southwest, potentially owing to the higher elevation in this area providing sufficient mountainous area wherein this species can move and adapt to local environmental conditions.

Climate change is recognized as a major driver of biodiversity loss [60]. Certain species may adjust their geographical distributions to match optimal environmental ranges as a means of adapting to these changing conditions [11]. However, the mobility of *C. jansoni* is limited by its large body size and long prolegs, significantly restricting its distribution, as it is likely unable to disperse over longer distances [29,30,32,33]. These biological traits suggest that *C. jansoni* depends on stable microhabitats for survival rather than relying on shifts in species distribution. This assumption is supported by the minimal shifts in distance predicted between *C. jansoni* centroids across various future climate change scenarios and periods. Similar conclusions have been drawn in other studies [37].

Ongoing climate change poses a significant threat to various species, particularly insects and those with narrow geographic distributions, which face a prominent risk of extinction [12,37]. Strengthened conservation measures are urgently required to ensure the protection of *C. jansoni*.

### 4.4. Implications for Biological Conservation

The results showed that only a limited subset of the highly suitable areas for *C. jansoni* in China are currently located within national nature reserves, while most suitable habitats lack effective protection (Figure 12 and Figure 13). Protected areas are recognized as effective approaches to preserving species’ habitats and biodiversity [61,62,63,64]. Given the close association between existing protected areas and suitable habitats for *C. jansoni*, establishing further nature reserves is essential to address protection gaps.

Effective management of protected areas is as critical as their establishment. Minimizing and eliminating habitat loss, fragmentation, and degradation caused by various human activities are important components of management. It is widely known that habitat transformation (including three forms: habitat loss, fragmentation, and degradation) is also a key driver of global ecosystem decline [65]. To halt biodiversity loss and mitigate the impacts of climate change, it is essential to significantly reduce the rate of habitat conversion and implement large-scale habitat restoration projects [65]. While significant research is still needed, efforts targeting one form of habitat transformation are expected to provide indirect benefits for the other two forms as well [65].

Specific and targeted measures are essential for optimizing habitat management. Artificial light significantly increases mortality rates in adult *C. jansoni* because of its strong phototactic behavior. Artificial light not only impacts *C. jansoni* but also affects other insects, amphibians, fish, birds, bats, and other animals, posing significant threats to biodiversity [66,67]. Light pollution is an intensifying concern that should be prioritized in conservation strategies. It can be diminished or minimized through the implementation of nature-friendly lighting design and management practices. Artificial light at night is easy to ameliorate and leaves behind no residual effects when compared to other anthropogenic habitat disturbances.

## Data Availability

The original contributions presented in the study are included in the article; further inquiries can be directed to the corresponding authors.

## Supplementary Materials

The following supporting information can be downloaded at https://www.mdpi.com/article/10.3390/insects15121012/s1: Figure S1: Optimized MaxEnt model ROC curves, averaged from replicate runs. The red and blue lines respectively denote the MaxEnt model fit for the training and testing datasets; Figure S2: Jackknife method-based analyses of environmental variable importance; Figure S3: The predicted distributions of *C. jansoni* under different future climate scenarios. (A) 2021–2040-SSP245; (B) 2041–2060-SSP245; (C) 2061–2080-SSP245; (D) 2081–2100-SSP245; Figure S4: The predicted distributions of *C. jansoni* under different future climate scenarios. (A) 2021–2040-SSP370; (B) 2041–2060-SSP370; (C) 2061–2080-SSP370; (D) 2081–2100-SSP370; Table S1: Environmental variables used for species distribution model construction; Table S2: Evaluation parameters for individual Biomod2 models based on 10 different modeling algorithms and their corresponding ensemble models; Table S3: Suitable areas for *C. jansoni* under different climate scenarios and changes in *C. jansoni* distribution based on binary classification under future climate scenarios in comparison to current climatic conditions (Units 103 km^2^). “Suitable habitat”, “Range Expansion”, and “Range Contraction” denote the proportions of change in suitable areas relative to current conditions. The “No occupancy” percentage corresponds to the proportion of unchanged areas unsuitable relative to current conditions. The “No change” percentage corresponds to the proportion of unchanged areas suitable relative to current conditions; Table S4: National nature reserves in China located within highly suitable areas for *C. jansoni*.

## References

[1] Masson-Delmotte V., Zhai P., Pirani A., Connors S.L., Péan C., Berger S., Caud N., Chen Y., Goldfarb L., Gomis M.I., IPCC (2021). Climate Change 2021: The Physical Science Basis. Contribution of Working Group I to the Sixth Assessment Report of the Intergovernmental Panel on Climate Change.

[2] Armstrong McKay D.I., Staal A., Abrams J.F., Winkelmann R., Sakschewski B., Loriani S., Fetzer I., Cornell S.E., Rockström J., Lenton T.M. (2022). Exceeding 1.5 °C global warming could trigger multiple climate tipping points. Science.

[3] Parmesan C. (2006). Ecological and evolutionary responses to recent climate change. Annu. Rev. Ecol. Evol. Syst..

[4] Chen I.C., Hill J.K., Ohlemüller R., Roy D.B., Thomas C.D. (2011). Rapid range shifts of species associated with high levels of climate warming. Science.

[5] Dore M.H.I. (2005). Climate change and changes in global precipitation patterns: What do we know?. Environ. Int..

[6] Li J.Y., Thompson D.W.J. (2021). Widespread changes in surface temperature persistence under climate change. Nature.

[7] Liu Z.H., Wu G.C. (2022). Quantifying the precipitation–temperature relationship in China during 1961–2018. Int. J. Climatol..

[8] Malhi Y., Franklin J., Seddon N., Solan M., Turner M.G., Field C.B., Knowlton N. (2020). Climate change and ecosystems: Threats, opportunities and solutions. Phil. Trans. R. Soc. B.

[9] Weiskopf S.R., Rubenstein M.A., Crozier L.G., Gaichas S., Griffis R., Halofsky J.E., Hyde K.J.W., Morelli T.L., Morisette J.T., Muñoz R.C. (2020). Climate change effects on biodiversity, ecosystems, ecosystem services, and natural resource management in the United States. Sci. Total Environ..

[10] Halsch C.A., Shapiro A.M., Fordyce J.A., Nice C.C., Thorne J.H., Waetjen D.P., Forister M.L. (2021). Insects and recent climate change. Proc. Natl. Acad. Sci. USA.

[11] Poloni R., Iannella M., Fusco G., Fattorini S. (2022). Conservation biogeography of high-altitude longhorn beetles under climate change. Insect Conserv. Diver..

[12] Moguel-Cárdenas L.I., León-Cortés J.L., Rodríguez-Aguilar O., Castillo-Vera A., Islebe G.A. (2024). Climate-driven change and conservation of threatened satyrine butterflies in cloud forests of southern Mexico. J. Insect Conserv..

[13] Kellermann V., van Heerwaarden B. (2019). Terrestrial insects and climate change: Adaptive responses in key traits. Physiol. Entomol..

[14] Harvey J.A., Tougeron K., Gols R., Heinen R., Abarca M., Abram P.K., Basset Y., Berg M., Boggs C., Brodeur J. (2023). Scientists’ warning on climate change and insects. Ecol. Monogr..

[15] Su J., Liu W.J., Hu F.C., Miao P.P., Xing L.X., Hua Y. (2023). The distribution pattern and species richness of scorpionflies (Mecoptera: Panorpidae). Insects.

[16] Elith J., Leathwick J.R. (2009). Species distribution models: Ecological explanation and prediction across space and time. Annu. Rev. Ecol. Evol. Syst..

[17] Rathore M.K., Sharma L.K. (2023). Efficacy of species distribution models (SDMs) for ecological realms to ascertain biological conservation and practices. Biodivers. Conserv..

[18] Guisan A., Tingley R., Baumgartner J.B., Naujokaitis-Lewis I., Sutcliffe P.R., Tulloch A.I.T., Regan T.J., Brotons L., Mcdonald-Madden E., Mantyka-Pringle C. (2013). Predicting species distributions for conservation decisions. Ecol. Lett..

[19] Singh A.P., De K., Uniyal V.P., Sathyakumar S. (2024). Unveiling of climate change-driven decline of suitable habitat for Himalayan bumblebees. Sci. Rep..

[20] Martínez-Minaya J., Cameletti M., Conesa D., Pennino M.G. (2018). Species distribution modeling: A statistical review with focus in spatio-temporal issues. Stoch. Environ. Res. Risk Assess..

[21] Elith J., Phillips S.J., Hastie T., Dudík M., Chee Y.E., Yates C.J. (2011). A statistical explanation of MaxEnt for ecologists. Divers. Distrib..

[22] Martínez-López O., Koch J.B., Martínez-Morales M.A., Navarrete-Gutiérrez D., Enríquez E., Vandame R. (2021). Reduction in the potential distribution of bumble bees (Apidae: *Bombus*) in Mesoamerica under different climate change scenarios: Conservation implications. Glob. Change Biol..

[23] Huang M.J., Hughes A.C., Xu C.Y., Miao B.G., Gao J., Peng Y.Q. (2022). Mapping the changing distribution of two important pollinating giant honeybees across 21,000 years. Glob. Ecol. Conserv..

[24] Wang X.Y., Li Z.S., Zhang L.J., Wang Y.L., Liu Y., Ma Y.S. (2024). The optimized Maxent model reveals the pattern of distribution and changes in the suitable cultivation areas for *Reaumuria songarica* being driven by climate change. Ecol. Evol..

[25] Thuiller W. (2003). BIOMOD: Optimising predictions of species distributions and projecting potential future shifts under global change. Glob. Change Biol..

[26] Araújo M.B., Pearson R.G., Thuiller W., Erhard M. (2005). Validation of species–climate impact models under climate change. Glob. Change Biol..

[27] Guo Y.L., Zhao Z.F., Qiao H.J., Wang R., Wei H.Y., Wang L.K., Gu W., Li X. (2020). Challenges and development trend of species distribution model. Adv. Earth Sci..

[28] Young R.M. (1989). Euchirinae (Coleoptera: Scarabaeidae) of the world: Distribution and taxonomy. Coleopt. Bull..

[29] Chen L., Xiong H.L., Li Z.L., Li Y.H., Wang Y. (2016). Preliminary observation on morphology and life habit of *Cheirotonus jansoni*. Hubei Agric. Sci..

[30] Xu Z.H., Xiao Y.H. (2019). The first record of *Cheirotonus jansoni* in Shiyan city, Hubei province. Hubei For. Sci. Technol..

[31] Liu S.L., Liu L.J., Wang D., Wu F.S., Zhou S.Z. (2022). A female description of *Cheirotonus jansoni* (Coleoptera, Euchiridae) from China. Hubei Agric. Sci..

[32] Zhao L. (2022). Giant insect in tree holes-*Cheirotonus jansoni*. Knowl. Is Power.

[33] Zhang W.W. (2013). Euchiridae: “gibbon” in insect world. Newton.

[34] Wu T.W., Lu Y.X., Fang Y.J., Xin X.G., Li L., Li W.P., Jie W.H., Zhang J., Liu Y.M., Zhang L. (2019). The Beijing Climate Center Climate System Model (BCC-CSM): The main progress from CMIP5 to CMIP6. Geosci. Model Dev..

[35] Zhou T.J., Chen Z.M., Chen X.L., Zuo M., Jiang J., Hu S. (2021). Interpreting IPCC AR6: Future global climate based on projection under scenarios and on near-term information. Climate Change Res..

[36] Merow C., Smith M.J., Silander J.A. (2013). A practical guide to MaxEnt for modeling species’ distributions: What it does, and why inputs and settings matter. Ecography.

[37] Liu T., Liu H.Y., Tong J.B., Yang Y.X. (2022). Habitat suitability of neotenic net-winged beetles (Coleoptera: Lycidae) in China using combined ecological models, with implications for biological conservation. Divers. Distrib..

[38] Ji W., Han K., Lu Y.Y., Wei J.F. (2020). Predicting the potential distribution of the vine mealy bug, *Planococcus ficus* under climate change by MaxEnt. Crop Prot..

[39] Liao J., Wang H.J., Xiao S.J., Guan Z.Y., Zhang H.M., Dumont H.J., Han B.P. (2022). Modeling and prediction of the species’ range of *Neurobasis chinensis* (Linnaeus, 1758) under climate change. Biology.

[40] Sun Z.X., Ye H.C., Huang W.J., Qimuge E., Bai H.Q., Nie C.J., Lu L.H., Qian B.X., Wu B. (2023). Assessment on potential suitable habitats of the grasshopper *Oedaleus decorus asiaticus* in north China based on MaxEnt modeling and remote sensing data. Insects.

[41] Cobos M.E., Peterson A.T., Barve N., Osorio-Olvera L. (2019). kuenm: An R package for detailed development of ecological niche models using Maxent. PeerJ.

[42] Phillips S.J., Anderson R.P., Dudík M., Schapire R.E., Blair M.E. (2017). Opening the black box: An open-source release of Maxent. Ecography.

[43] Li D.X., Li Z.X., Liu Z.W., Yang Y.J., Khoso A.G., Wang L., Liu D.G. (2023). Climate change simulations revealed potentially drastic shifts in insect community structure and crop yields in China’s farmland. J. Pest Sci..

[44] Fielding A.H., Bell J.F. (1997). A review of methods for the assessment of prediction errors in conservation presence/absence models. Environ. Conserv..

[45] Thuiller W., Lafourcade B., Engler R., Araújo M.B. (2009). BIOMOD—A platform for ensemble forecasting of species distributions. Ecography.

[46] Cohen J. (1960). A coefficient of agreement of nominal scales. Educ. Psychol. Meas..

[47] Allouche O., Tsoar A., Kadmon R. (2006). Assessing the accuracy of species distribution models: Prevalence, kappa and the true skill statistic (TSS). J. Appl. Ecol..

[48] Aidoo O.F., Souza P.G.C., da Silva R.S., Santana P.A., Picanço M.C., Kyerematen R., Sètamou M., Ekesi S., Borgemeister C. (2022). Climate-induced range shifts of invasive species (*Diaphorina citri* Kuwayama). Pest Manag. Sci..

[49] Zhao G.H., Cui X.Y., Sun J.J., Li T.T., Wang Q., Ye X.Z., Fan B.G. (2021). Analysis of the distribution pattern of Chinese *Ziziphus jujuba* under climate change based on optimized biomod2 and MaxEnt models. Ecol. Indic..

[50] Araújo M.B., New M. (2007). Ensemble forecasting of species distributions. Trends Ecol. Evol..

[51] Pearson R.G., Dawson T.P. (2003). Predicting the impacts of climate change on the distribution of species: Are bioclimate envelope models useful?. Glob. Ecol. Biogeogr..

[52] Wang X.S., Chen R. (2021). Wuyi Mountain -- Adventure in the legendary reserve. Knowl. Is Power.

[53] Wang Z.Z., Zhang M., Zhao X.B., Xie J.M., Peng Y.G., Sheldon F.H., Zou F.S. (2024). The Nanling Mountains of southern China played a variable role as a barrier and refuge for birds depending upon landscape structure and timing of events. J. Avian Biol..

[54] Bui V.B., Ziegler T., Bonkowski M. (2020). Morphological traits reflect dung beetle response to land use changes in tropical karst ecosystems of Vietnam. Ecol. Indic..

[55] Méndez-Rojas D.M., Cultid-Medina C., Escobar F. (2021). Influence of land use change on rove beetle diversity: A systematic review and global meta-analysis of a mega-diverse insect group. Ecol. Indic..

[56] Bellaver J.M.F., de Souza Lima-Ribeiro M., Hoffmann D., Romanowski H.P. (2022). Rare and common species are doomed by climate change? A case study with neotropical butterflies and their host plants. J. Insect Conserv..

[57] Li A.N., Wang J.W., Wang R.L., Yang H., Yang W., Yang C.P., Jin Z. (2020). MaxEnt modeling to predict current and future distributions of *Batocera lineolata* (Coleoptera: Cerambycidae) under climate change in China. Ecoscience.

[58] Guo P.C., Zhao X., Shi J.K., Huang J.C., Tang J., Zhang R.R., Chen J., Wang Q.F., Zeng J.Y. (2020). The influence of temperature and precipitation on the vegetation dynamics of the tropical island of Hainan. Theor. Appl. Climatol..

[59] Cronk Q. (1997). Islands: Stability, diversity, conservation. Biodivers. Conserv..

[60] Habibullah M.S., Din B.H., Tan S.H., Zahid H. (2022). Impact of climate change on biodiversity loss: Global evidence. Environ. Sci. Pollut. Res..

[61] Liu X.Y., Li X.S., Zhao C.Y., Li F.F., Zhu J.F., Ji W.J. (2021). Simulation of potential suitable distribution of *Bhutanitis thaidina* and its gap analysis of National Nature Reserves in China under climate change scenarios. J. Environ. Entomol..

[62] Xue D.Y., Jiang M.K. (1995). Contributions of Nature Reserves in China to biodiversity conservation. J. Nat. Resour..

[63] Gray C.L., Hill S.L., Newbold T., Hudson L.N., Borger L., Contu S., Hoskins A.J., Ferrier S., Purvis A., Scharlemann J.P. (2016). Local biodiversity is higher inside than outside terrestrial protected areas worldwide. Nat. Commun..

[64] Watson J.E., Dudley N., Segan D.B., Hockings M. (2014). The performance and potential of protected areas. Nature.

[65] Banks-Leite C., Ewers R.M., Folkard-Tapp H., Fraser A. (2020). Countering the effects of habitat loss, fragmentation, and degradation through habitat restoration. One Earth.

[66] Rich C., Longcore T. (2006). Ecological Consequences of Artificial Night Lighting.

[67] Hölker F., Wolter C., Perkin E.K., Tockner K. (2010). Light pollution as a biodiversity threat. Trends Ecol. Evol..

