# Supplementary material for: Predicting the Potential Distribution of Cheirotonus jansoni (Coleoptera: Scarabaeidae) Under Climate Change
Source: Insects. 2024 Dec 20;15(12):1012. doi: 10.3390/insects15121012 (PMC11677015; doi:10.3390/insects15121012)
Supplement: Supplementary file 1 [file insects-15-01012-s001.zip › insects-3303530-supplementary.pdf]

insects

Supplementary Materials

Predicting the Potential Distribution of *Cheirotonus jansoni* (Coleoptera: Scarabaeidae) Under Climate Change

Yali Yu [1,2] and Zhiqiang Li [1,2,*]

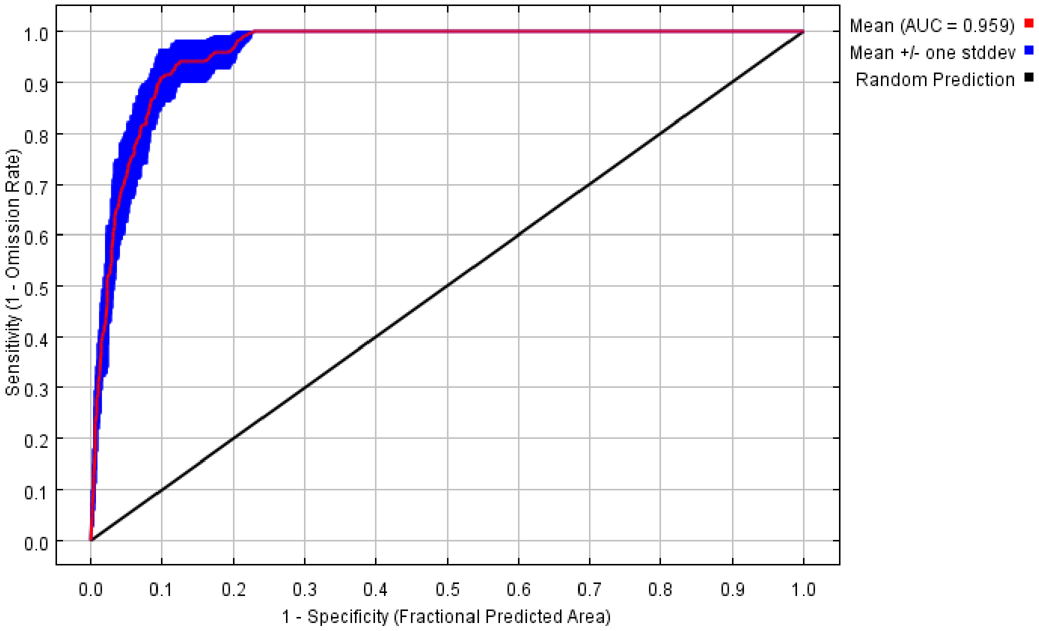

Figure S1. Optimized MaxEnt model ROC curves, averaged from replicate runs. The red and blue lines respectively denote the MaxEnt model fit for the training and testing datasets.

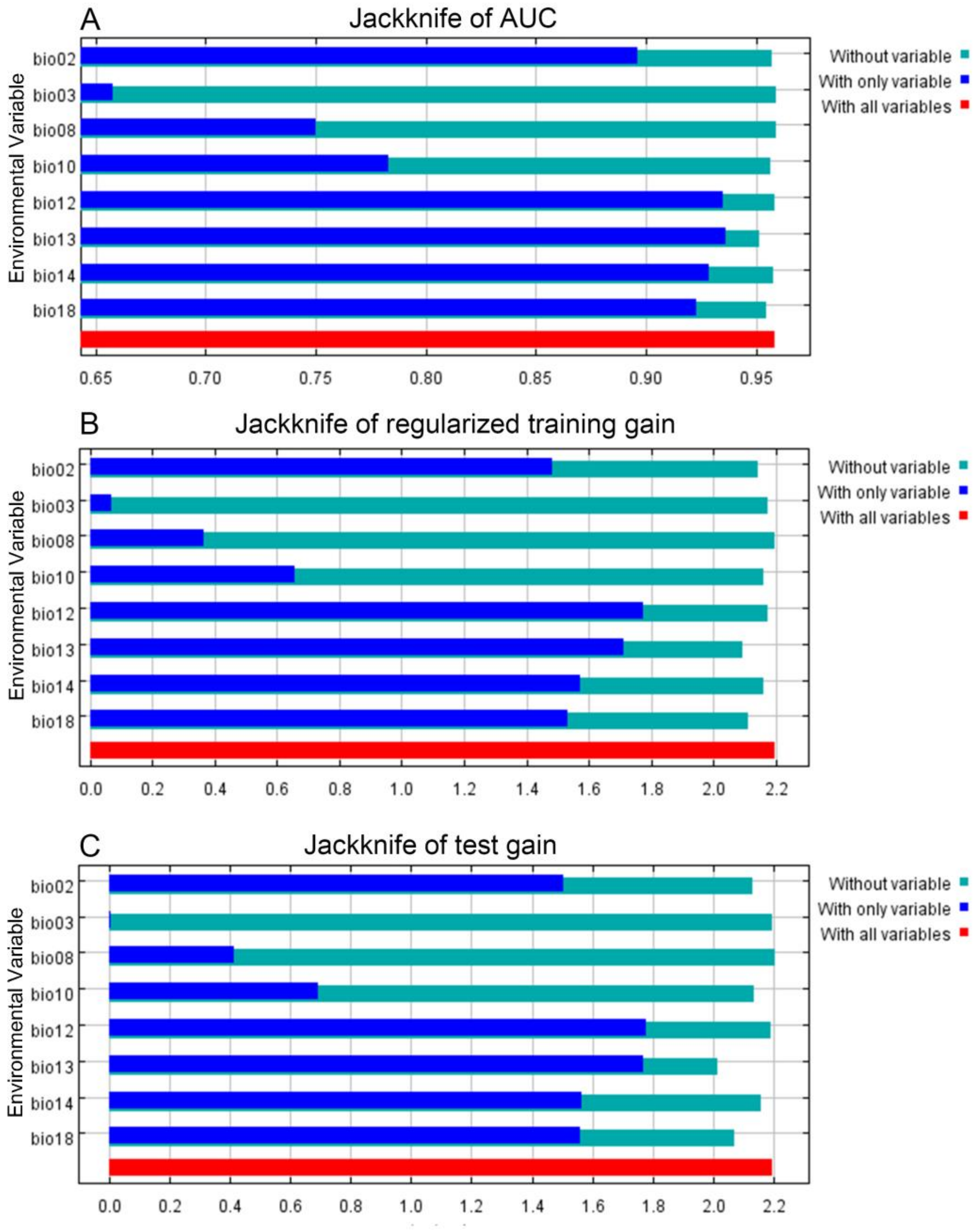

Figure S2: Jackknife method-based analyses of environmental variable importance;

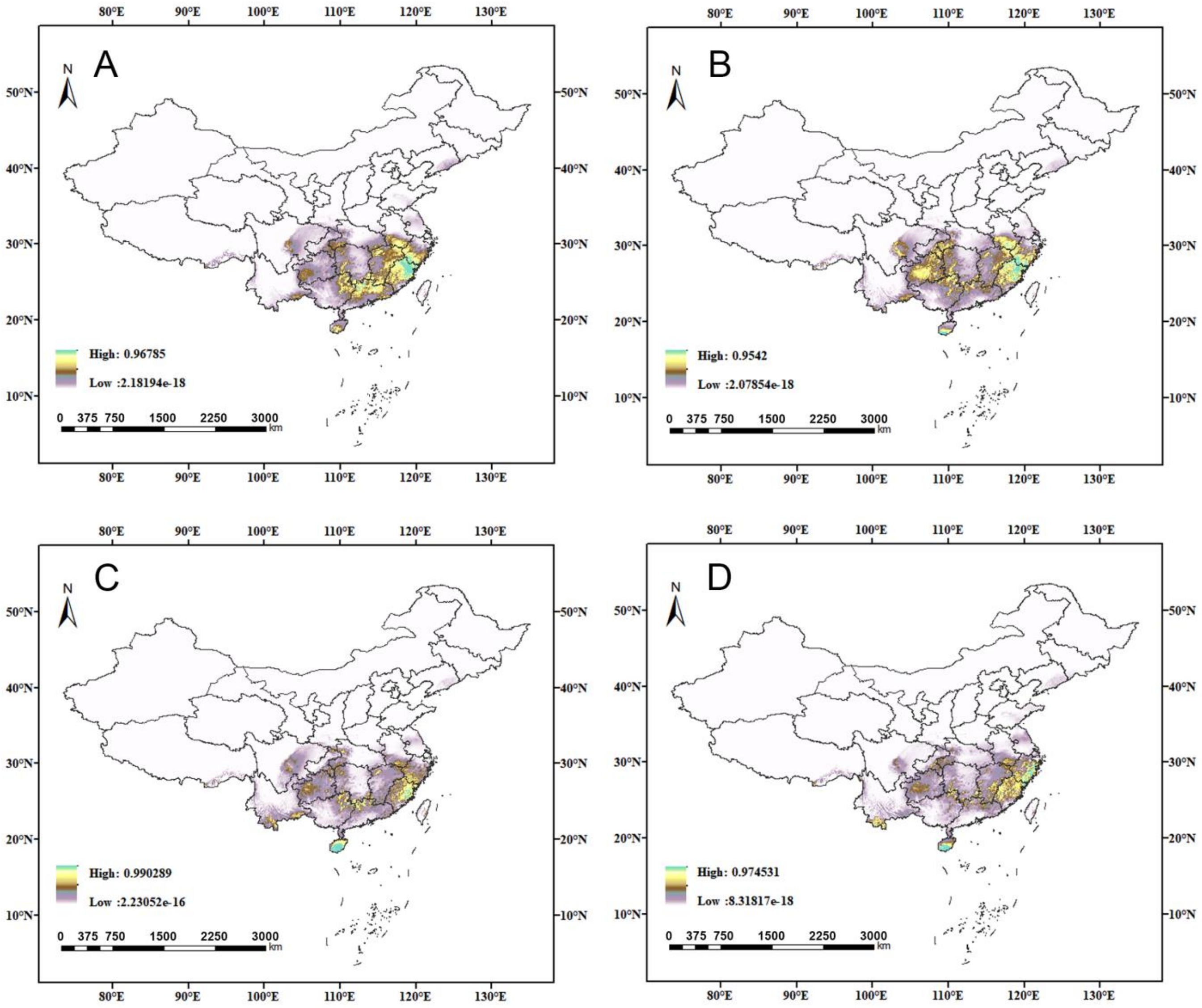

Figure S3. The predicted distributions of *C. jansoni* under different future climate scenarios. (A) 2021–2040-SSP245; (B) 2041–2060-SSP245; (C) 2061–2080-SSP245; (D) 2081–2100-SSP245;.

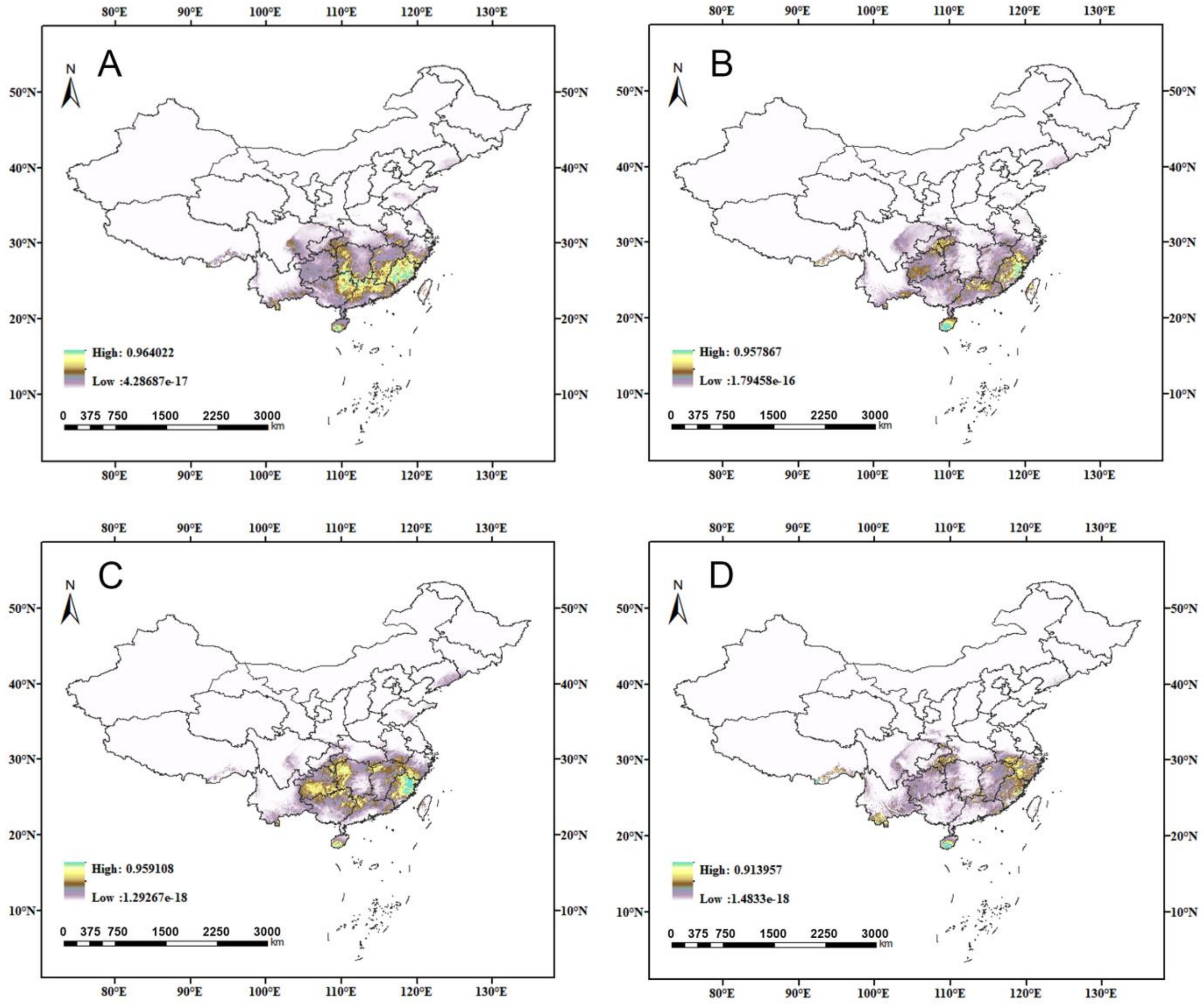

Figure S4. The predicted distributions of *C. jansoni* under different future climate scenarios. (A) 2021–2040-SSP370; (B) 2041–2060-SSP370; (C) 2061–2080-SSP370; (D) 2081–2100-SSP370;.

Table S1. Environmental variables used for species distribution model construction;.

Variables	Description	Units	Percent contribution	Permutation importance
bio01	Annual average temperature	°C	–	–
bio02*	Average variation in daytime temperature	°C	53.9	12.5
bio03*	Isothermality (bio02/bio07) (×100)	–	2	1
bio04	Seasonality of temperature (standard deviation×100)	–	–	–
bio05	Highest temperature of the hottest month	°C	–	–
bio06	Lowest temperature of the coldest month	°C	–	–
bio07	Annual temperature variation (bio05–bio06)	°C	–	–
bio08*	Average temperature of the rainy quarter months	°C	4.4	1.5
bio09	Average temperature of the driest quarter months	°C	–	–
bio10*	Average temperature of the hottest quarter months	°C	4	10.9
bio11	Average temperature of the coldest quarter months	°C	–	–
bio12*	Annual Precipitation	mm	14.7	29.9
bio13*	Precipitation of the rainiest month	mm	5.2	36.3
bio14*	Precipitation of driest month	mm	5	1.4

bio15	Precipitation Seasonality (coefficient of variation)	–	–	–
bio16	Precipitation of the rainiest quarter months	mm	–	–
bio17	Precipitation of the driest quarter months	mm	–	–
bio18*	Precipitation of the hottest quarter months	mm	10.8	6.5
bio19	Precipitation of the coldest quarter months	mm	–	–

Table S2. Evaluation parameters for individual Biomod2 models based on 10 different modeling algorithms and their corresponding ensemble models.

data	KAPPA	TSS	AUC	data	KAPPA	TSS	AUC
GLM.PA1	0.677	0.883	0.971	GLM.PA2	0.65	0.742	0.871
GBM.PA1	0.748	0.941	0.985	GBM.PA2	0.788	0.941	0.987
GAM.PA1	0.721	0.927	0.978	GAM.PA2	0.778	0.936	0.984
CTA.PA1	0.441	0.827	0.913	CTA.PA2	0.429	0.82	0.91
ANN.PA1	0.671	0.923	0.966	ANN.PA2	0.705	0.926	0.974
SRE.PA1	0.512	0.611	0.805	SRE.PA2	0.509	0.61	0.805
FDA.PA1	0.674	0.848	0.961	FDA.PA2	0.662	0.864	0.969
MARS.PA1	0.661	0.884	0.969	MARS.PA2	0.674	0.881	0.969
RF.PA1	0.988	0.998	1	RF.PA2	0.982	0.993	1
MAXENT.Phillips.PA1	0.841	0.87	0.977	MAXENT.Phillips.PA2	0.746	0.88	0.977
Ensemble models.ca	0.832	0.917	0.993	Ensemble models.wm	0.671	0.913	0.986

Note: "PA1" denotes the first time when selecting pseudoabsence points; "PA2" denotes the second time when selecting pseudoabsence points; "ca" denotes the committee averaging method for the ensemble models; "wm" indicates the weighted mean of probabilities for the ensemble models.

Table S3. Suitable areas for *C. jansoni* under different climate scenarios and changes in *C. jansoni* distribution based on binary classification under future climate scenarios in comparison to current climatic conditions (Units 103 km^2). "Suitable habitat", "Range Expansion", and "Range Contraction" denote the proportions of change in suitable areas relative to current conditions. The "No occupancy" percentage corresponds to the proportion of unchanged areas unsuitable relative to current conditions. The "No change" percentage corresponds to the proportion of unchanged areas suitable relative to current conditions.

Climate scenario	Suitable habitat (area & percentage)	Range Expansion (area & percentage)	No Occupancy (absence in both) (area & percentage)	No Change (presence in both) (area & percentage)	Range Contraction (area & percentage)
Current	1044.19 (–)	(–)	(–)	(–)	(–)
2021-2040-SSP245	903.74 （-0.135）	50.28 （0.048）	8505.53 （0.994）	853.47 （0.817）	190.72 （0.183）
2041-2060-SSP245	938.28 （-0.101）	60.12 （0.058）	8495.69 （0.993）	878.16 （0.841）	166.03 （0.159）
2061-2080-SSP245	760.90 （-0.271）	71.87 （0.069）	8483.94 （0.992）	689.03 （0.660）	355.16 （0.340）
2081-2100-SSP245	694.84 （-0.335）	65.98 （0.063）	8489.83 （0.992）	628.86 （0.602）	415.33 （0.398）
2021-2040-SSP370	927.82 （-0.111）	87.95 （0.084）	8467.86 （0.990）	839.86 （0.804）	204.33 （0.196）
2041-2060-SSP370	551.62 （-0.472）	28.37 （0.027）	8527.44 （0.997）	523.25 （0.501）	520.94 （0.499）
2061-2080-SSP370	849.39 （-0.187）	75.79 （0.073）	8480.02 （0.991）	773.61 （0.741）	270.58 （0.259）
2081-2100-SSP370	403.08 （-0.614）	50.69 （0.049）	8505.12 （0.994）	352.38 （0.337）	691.81 （0.663）

Table S4: National nature reserves in China located within highly suitable areas for *C. jansoni*.

No.	Name	Province	Type	Area (ha)
1	Anhui Guniujiang National Nature Reserve	Anhui	Forest ecological reserve	6713.0
2	Jiangxi Jiulingshan National Nature Reserve	Jiangxi	Forest ecological reserve	11541.0
3	Jiangxi Guanshan National Nature Reserve	Jiangxi	Forest ecological reserve	11500.5
4	Jiangxi Jinggangshan National Nature Reserve	Jiangxi	Forest ecological reserve	21499.0
5	Jiangxi Qiyunshan National Nature Reserve	Jiangxi	Forest ecological reserve	17105.0
6	Jiangxi Jiulianshan National Nature Reserve	Jiangxi	Forest ecological reserve	13411.6
7	Jiangxi Matou Mountain National Nature Reserve	Jiangxi	Forest ecological reserve	13866.53
8	Jiangxi Yangjifeng National Nature Reserve	Jiangxi	Wildlife Sanctuaries	10946.0
9	Jiangxi Wuyi Mountain National Nature Reserve	Jiangxi	Forest ecological reserve	16007.0
10	Zhejiang Gutian Mountain National Nature Reserve	Zhejiang	Wildlife Sanctuaries	8108.0
11	Zhejiang Jiulongshan National Nature Reserve	Zhejiang	Forest ecological reserve	5525.0
12	Zhejiang Fengyangshan-Naishanzu National Nature Reserve	Zhejiang	Forest ecological reserve	26051.5
13	Zhejiang Wuyanling National Nature Reserve	Zhejiang	Forest ecological reserve	18861.5
14	Fujian Wuyi Mountain National Nature Reserve	Fujian	Forest ecological reserve	56527.0
15	Fujian Minjiang Yuan National Nature Reserve	Fujian	Forest ecological reserve	13022.0
16	Fujian Junzifeng National Nature Reserve	Fujian	Forest ecological reserve	18060.5
17	Fujian Longqishan National Nature Reserve	Fujian	Forest ecological reserve	15693.0
18	Fujian Xiongjiang Huangchu Forest National Nature Reserve	Fujian	Forest ecological reserve	12513.3
19	Fujian Tingjiangyuan National Nature Reserve	Fujian	Forest ecological reserve	10379.7
20	Fujian Liangye Mountain National Nature Reserve	Fujian	Wildlife Sanctuaries	14365.0
21	Fujian Meihuashan National Nature Reserve	Fujian	Forest ecological reserve	22168.0
22	Fujian Tianbaoyan National Nature Reserve	Fujian	Forest ecological reserve	11015.0
23	Daiyun Mountain National Nature Reserve	Fujian	Forest ecological reserve	13472.4
24	Hunan Huangsang National Nature Reserve	Hunan	Forest ecological reserve	12590.0
25	Hunan Shunhuang Mountain National Nature Reserve	Hunan	Forest ecological reserve	3533.0
26	Hunan Jintongshan National Nature Reserve	Hunan	Forest ecological reserve	18466.0
27	Hunan Dupangling National Nature Reserve	Hunan	Forest ecological reserve	20066.0
28	Hunan Mangshan National Nature Reserve	Hunan	Forest ecological reserve	19833.0
29	Hunan Bamianshan National Nature Reserve	Hunan	Forest ecological reserve	10974.0
30	Yuanling Taoyuandong National Nature Reserve	Hunan	Forest ecological reserve	23786.0
31	Guangxi Jiuwan Mountain National Nature Reserve	Guangxi	Forest ecological reserve	25212.8
32	Guangxi Yuanbaoshan National Nature Reserve	Guangxi	Wetland Ecological Reserve	4159.0
33	Guangxi Huaping National Nature Reserve	Guangxi	Forest ecological reserve	17400.0
34	Guangxi Mao'er Mountain National Natural Reserve	Guangxi	Forest ecological reserve	17008.5
35	Guangxi Qianjiadong National Natural Reserve	Guangxi	Forest ecological reserve	12231.0
36	Guangxi Dayao Mountain National Natural Reserve	Guangxi	Forest ecological reserve	24907.3
37	Guangxi Qichong National Natural Reserve	Guangxi	Forest ecological reserve	13023.7
38	Guangdong Nanling Mountain National Nature Reserve	Guangdong	Forest ecological reserve	50000.0
39	Guangdong Shimentai National Nature Reserve	Guangdong	Forest ecological reserve	33555.0
40	Guangdong Chebaling National Nature Reserve	Guangdong	Forest ecological reserve	7545.0
41	Hainan Jianfengling National Nature Reserve	Hainan	Forest ecological reserve	20170.0
42	Hainan Bawangling National Nature Reserve	Hainan	Wildlife Sanctuaries	29980.0
43	Hainan Wuzhishan National Nature Reserve	Hainan	Forest ecological reserve	13435.9
44	Hainan Diaoluoshan National Nature Reserve	Hainan	Forest ecological reserve	18389.0
